# Knowledge and attitude towards epilepsy among rural residents in southern Ethiopia: a cross-sectional study

**DOI:** 10.1186/s12889-021-10467-8

**Published:** 2021-02-27

**Authors:** Alemayehu Molla, Birhanie Mekuriaw, Endashaw Habtamu, Moges Mareg

**Affiliations:** 1 Department of Psychiatry, College of Health and Medical Science, Dilla University, Dilla, Ethiopia; 2 Department of Reproductive health, College of Health and Medical Science, Dilla University, Dilla, Ethiopia

**Keywords:** Attitude, Epilepsy, Ethiopia, Knowledge, Rural communities

## Abstract

**Background:**

Although epilepsy is one of the most prevalent neurological problems, it is highly surrounded by stigma and prejudice, which results in negative attitude towards the illness. Due to numerous misconceptions and beliefs attributed towards epilepsy, most people in rural communities have poor understanding and perception about epilepsy. Studying knowledge and attitude of this major neurologic problem among rural residents is crucial to add knowledge and show area of interventions. Therefore, the aim of this study was to assess the knowledge and attitude towards Epilepsy among rural residents in Ethiopia.

**Methods:**

This was a community based cross-sectional study conducted in rural parts of Gedeo zone, Southern Ethiopia. A total of 732 randomly selected adult residents were interviewed using a pre-tested questionnaire. The collected data were entered to Epi-data version 3.1 and analyzed using SPSS version 20. Descriptive statistics and logistic regressions were performed. Multivariable binary logistic regression analysis was conducted to determine the presence of a statistically significant association between explanatory variables and outcome variables at corresponding 95% CI.

**Results:**

The magnitude of poor knowledge and unfavorable attitude towards epilepsy were 27.0 and 51.6%, respectively. Participants who can’t read and write, having stigma related to epilepsy, participants who did not live with epileptic patients; unfavorable attitude and age were factors associated with poor knowledge towards epilepsy. On the other hand, Stigma related to epilepsy, poor knowledge, age and perceiving epilepsy as a God punishment for sinful activities were variables significantly associated with unfavorable attitude of epilepsy.

**Conclusions:**

There is a gap regarding the knowledge and attitude towards epilepsy among community residents in southern Ethiopia. This demonstrates a need for community educational program regarding epilepsy which can increase community awareness particularly in rural areas to decrease stigma and negative beliefs towards epilepsy.

## Background

Neuropsychiatry disorders account more than a quarter of the global burden of diseases [1]. Epilepsy is one of the known neurological disorders characterized by repeated seizures and with at least two unprovoked seizure episodes [2]. The impact of epilepsy in terms of poor quality of life, morbidity, mortality, and stigma varies worldwide depending on economic, cultural, and health backgrounds of communities [3]. Epilepsy remains a major public health problem with its social, cultural, psychological and economic effects [4, 5].

Misperceptions and indigenous beliefs about the causes and inheritability of epilepsy make the lives of people with epilepsy (PWE) more difficult, particular in low and middle income countries (LMIC) [6]. Besides, stigma related to epilepsy is common in developing countries, and its social, psychological and economic consequences become a major public health problems [4, 7]. The religious and cultural beliefs have wide ranges of effects on treatment options and the adherence level among people with epilepsy [7]. Socio-cultural beliefs cause a negative impact to patients with epilepsy in many developing nations [8]. For instance, many people in developing countries believe that epilepsy is caused by witchcraft or evil spirits, and treatment should be through the use of traditional healing practice and religious leaders [8–10].

About 81% of PWE and their relatives affected from perceived stigma related to epilepsy in Ethiopia [11]. Such unfavorable attitudes and beliefs had serious negative social and psychological impact for PWE such as fear, and decrement in social interactions [12].

Traditional and supernatural perceptions also can have a great negative influence on the treatment delay and adherence of people with epilepsy. For instance, in developing countries, 60 to 90% of people with epilepsy receive no treatment due to negative beliefs regarding epilepsy [4, 13]. As a result, people with epilepsy are affected by a multitude of social, economic and psychological problems of stigmatization and lead to poor quality of life for people with epilepsy [14, 15].

It is important that investigation of the communities perception and attitude towards epilepsy helps to demystify individual and familial beliefs regarding the disease and to reduce the emotional impact of having a seizure in front of others [12, 16]. Although knowledge and attitudes regarding epilepsy were assessed in Ethiopia, their focus was among students and residents living in the towns [8, 17] and it is not well investigated among community residents. The current study aimed to assess community knowledge and attitude towards epilepsy among rural residents in Gedeo Zone, Southern Nation Nationalities and Peoples Region (SNNPR), Ethiopia.

Finding of the study will have valuable contribution in the current state of knowledge by providing appropriate evidences which will be important for decision makers, politicians, health personnel and future researchers.

## Methods and materials

### Study design

This was a community based cross sectional study conducted from March 1 to March 30, 2019.

### Study setting

The study was conducted in rural community residents of Gedeo zone residents. Gedeo is one of the eleven zones found in SNNPR with the administrative center of Dilla town located 360 km away from Addis Ababa (the capital city of Ethiopia). Gedeo zone has six rural districts and two administrative towns (Dilla and Yirgachefe). The 2007 census conducted by the central statistical agency of Ethiopia reported that the zone has a total population of 1, 086,768 with 532,516(49%) men and 554, 225 (51%) women [18]. More than 179,677 households were counted with the average 5 person living in each household and total of 276 health facilities were found in the zone [19]. People from multiple ethnic backgrounds are living in Gedeo zone. One special characteristics of this zone is that most of community residents use two leg motor bicycles which magnifies risk of head injury, one of the expected cause for epilepsy [20, 21].

### Study subjects

All permanent residents (living in the area for at least six months) with age 18 years and above and avail during data collection period were study subjects. Individuals who were severely ill during data collection time and unable to communicate were excluded from the study.

### Sampling size and procedure

Sample size was calculated by using a single population proportion formula; considering 66.5% of residents have poor practice towards epilepsy (study conducted in western parts of Ethiopia [8]) and margin of error 5%. After using design effect of 2 and inflating sample size by 10% to account for non- response rate, the final sample size was 755. Multistage sampling technique was applied to select study participants. In the first stage, 3 districts were selected out of six rural districts in Gedeo zone using lottery method. Then, random selections of 3 sub districts from each selected district was employed. The number of residents and households list for each of the selected sub district were obtained from corresponding district offices and health extension workers. Accordingly, the calculated sample size was proportionally allocated to the selected sub districts and one individual was interviewed from each household consecutively till the sample size was addressed.

### Data collection instruments

Data was collected by interview using pretested questionnaires. The contents of the questionnaire included independent variables like socio demographic characteristics, psychosocial factors, clinical variables and outcome variables knowledge and attitude towards Epilepsy. The outcome variables were assessed by knowledge and attitude related questions. Knowledge towards epilepsy was assessed using researcher prepared questions based on previous similar studies [17] and cultural context of study area and it has “yes” or “no” options towards epilepsy. It includes general information towards epilepsy, common manifestations and expected causes; in this case about 27% of participants reported as they had poor knowledge towards epilepsy. Attitude was assessed using 7-item questionnaires after modifying Kilifi epilepsy beliefs and attitude scale in our cultural context. The questionnaire includes attitude of respondents about employment opportunity, marital prospects, educational ability, believing epilepsy is result of punishment from God due to wrong doing, chance of cure by modern medicine and attitude of the respondents towards living with epileptic patient [8, 9, 22–24]. We used the following options for each question: 1 = strongly disagree, 2 = disagree, 3 = agree, 4 = strongly agree. We categorized participants as with favorable and unfavorable attitude based on the mean score which was 51.6.

Epilepsy stigma was assessed by using 24 item epilepsy stigma scales, participants were asked to choice suitable answer among following options: 1 = not at all, 2 = a little, 3 = a lot, 4 = totally. Finally all possible answers were summed and mean score was used to identify participants if they had stigma or not [25]. The internal consistence (cronbach alpha) of 24-item Epilepsy stigma scale in current study is 0.81.

### Data quality assurance

The questionnaire was first prepared in English and translated to local languages (Gedeo’fa and Amharic) and back translation to English was done to check its consistency. Data was collected by 9 BSc nurses and supervised by 3 MSc level psychiatry professionals. Two days training was given to data collectors and supervisors. Pre-test was done on 5% (n=38) of participants. Based on the finding of the pretest, the questionnaire was revised and the expressions of some contents were rephrased to make it easier to be understood by study subjects. The data collectors were supervised daily and the filled questionnaires were checked daily by the supervisors and investigators for its completeness.

### Data analysis procedure

The coded data was entered to Epi-data 3.1 and analyzed using SPSS version 20. Data was presented using frequencies and percentages. Bivariate binary logistic regression analysis was conducted to determine each independent variables and variables with *p*- value less than 0.2 were entered to multivariate analysis. Multivariate binary logistic regression analysis was performed to identify statistically associated variables with outcome variables. Finally, variables with *P* values less than 0.05 were considered statistically significant and strength of the association was presented by adjusted odds ratio with corresponding 95% C.I.

## Results

### Socio-demographic distribution of the respondents

A total of 732 participants were included in the study with the response rate of 97%. The mean age (±SD) of the respondents was 33(±11.3) years, with age range of 18–70 years. Among the total respondents, a majority were in age range of 18–28 years 316 (43.2%) and 467 (63.8%) were male participants. The largest proportion of the participants, 513 (70.1%) were protestant religion follower, and 632(86.3%) were Gedeo in their ethnicity (Table 1).

**Table 1:** Distribution of socio-demographic factors in rural Gedeo zone, Ethiopia, 2019 (*n* = 732)

Variable	Categories	Frequency	Percent (%)
Age	18–28 years	316	43.2
	29–39 years	231	31.6
	40–50 years	112	15.3
	> 50 years	73	10.0
Sex	Male	467	63.8
	Female	265	36.2
Religion	Protestant	513	70.1
	Orthodox	117	16.0
	Muslim	42	5.7
	Catholic	40	5.5
	Others^a^	20	2.7
Marital status	Married	513	70.1
	Single	147	20.1
	Widowed	35	4.8
	Divorced	25	3.4
	Separated	12	1.6
Ethnicity	Gedeo	632	86.3
	Oromo	45	6.1
	Amhara	28	3.8
	Gurage	20	2.7
	Others^b^	8	1.1
Educational level	Can’t read and write	196	26.8
	Primary	276	37.7
	Secondary	151	20.6
	Preparatory	51	7.0
	College and above	58	7.9
Occupational status	Government employed	96	13.1
	Farmer	232	31.7
	Merchant	105	14.3
	Private employed	116	15.8
	House wife	94	12.8
	Daily laborer	24	3.3
	Others ^c^	65	8.9
Distance to health center (km)	< 10 km	718	98.1
	≥10 km	14	1.9
Average monthly income	< 1539 ETB	538	73.5
	≥1539ETB	194	26.5

^a^Joba witness & no religion, ^b^Tigre, Wolaita & Silte, ^c^Jobless & Students

### Psychosocial and clinical related factors of respondents

Regarding the psychosocial characteristics of the respondents, 347 (47.4%) of participants reported stigma related to epilepsy and more than half reported that they have poor social support 451 (61.6%). About 54 (7.4%) respondents reported history of epilepsy, but only 12 (1.6%) of them got help for their problem **(**Table 2).

**Table 2:** Description of psychosocial and clinical factors among rural residents, in Gedeo zone 2019 (n = 732)

Variables	Categories	Frequency	Percent (%)
Stigma related to epilepsy	Yes	347	47.4
	No	385	52.6
Social support	Poor	451	61.6
	Moderate	249	34.0
	Strong	32	4.4
History of epilepsy	Yes	54	7.4
	No	678	92.6
Get help for illness	Yes	12	1.6
	No	720	98.4
Place of treatment	Health institutions	2	16.7
	Religious centers	7	58.3
	Traditional healers	3	25.0
Reason not to get help	No treatment	31	73.8
	No money	1	2.4
	Does not need treatment	10	23.8
Family history of epilepsy	Yes	61	8.3
	No	671	91.7
Family history of mental illness	Yes	76	10.4
	No	656	89.6
Previous history of head injury	Yes	58	7.9
	No	674	92.1

### Knowledge and attitude related factors towards epilepsy

Among participants, 198 (27%) of respondents had no information about epilepsy. Regarding to the source of information, large numbers 274 (37.4%) of them heard from their friends. Of the total participants, 49 (6.7%) reported as they were living with epileptic patients in one house and about 154 (21%) have history of witnessing seizure. Participants response regarding the cause of epilepsy showed that majorities 274 (37.4%) of them perceived that epilepsy is a punishment from the God for sin. Concerning to the symptoms of epilepsy, large numbers of participants 592(80.9%) reported convulsion as a symptom of epilepsy followed by loss of consciousness 407 (55.6%). Regarding to the overall attitude of participants towards epilepsy, about 378 (51.6%) had unfavorable attitude (Table 3).

**Table 3:** Distribution of Knowledge and Attitude related variables towards Epilepsy among rural residents, in Gedeo zone 2019 (n = 732)

Variables	Categories	Frequency	Percent (%)
Have information about epilepsy	Yes	534	73.0
	No	198	27.0
Source of information	Health professionals	150	20.5
	Media	90	12.3
	Friends	274	37.4
	Others	35	4.8
Live with Epileptic patient	Yes	49	6.7
	No	683	93.3
Witnessing seizure	Yes	154	21.0
	No	578	79.0
Perceiving epilepsy as punishment for sin	Yes	274	37.4
	No	458	62.6
Perceiving Substance is a cause for epilepsy	Yes	82	11.2
	No	650	88.8
Head injury as cause of epilepsy	Yes	212	29.0
	No	520	71.0
Convulsion as a symptoms of epilepsy	Yes	592	80.9
	No	140	19.1
Loss of consciousness as symptom of epilepsy	Yes	407	55.6
	No	325	44.4
Others as symptoms of epilepsy ^k^	Yes	34	4.6
	No	698	95.4
Attitude towards epilepsy	favorable attitude	354	48.4
	unfavorable attitude	378	51.6
I can marry epileptic patients	Agree	148	20.2
	Disagree	584	78.8
Epilepsy is curable	agree	488	66.7
	disagree	224	33.3
I can be a neighbor with epileptic patients	Agree	569	77.7
	Disagree	163	22.3
I can Employ epileptics patients	Agree	466	63.4
	Disagree	268	36.6

^k^- Unknown and irritability

### Factors associated with knowledge status towards epilepsy

In the multivariate logistic regression analysis, participants who cannot read and write, having stigma related to epilepsy, participants without history of living with epileptic patients, age and unfavorable attitude towards epilepsy were variables statistically associated with poor knowledge about epilepsy at *P*-value less than 0.05 (Table 4**)**.

**Table 4:** Bivariate and multivariate logistic regression analysis of associated factors for poor. Knowledge towards Epilepsy among rural Gedeo zone residents, 2019

Explanatory variables	Categories	Knowledge		COR, (95%CI)	AOR, (95%CI)
		Poor n(%)	Good n(%)		
Sex	Male	111 (15.2)	356 (48.6)	Referent	Referent
	Female	87 (11.9)	178 (24.3)	1.57,(1.12,4.31)	1.02 (0.68, 1.53)
Educational status	Cannot read and write	90 (12.3)	106 (14.5)	4.60, (2.15, 9.93)	6.34,(2.66, 15.10)**
	Primary	62 (8.5)	214 (29.3)	1.58, (0.73, 3.39)	1.45,(0.63, 3.34)
	Secondary	31 (4.2)	120 (16.4)	1.41, (0.62, 3.17)	1.69,(0.71, 4.07)
	Preparatory	6 (0.8)	45 (6.2)	0.73, (0.24, 2.20)	0.81,(0.25, 2.66)
	College and above	9 (1.2)	49 (6.7)	Referent	Referent
living with epileptic patients	Yes	2 (0.3)	47 (6.4)	Referent	Referent
	No	196 (26.8)	487 (66.5)	9.46 (2.28, 39.3)	11.15,(2.25, 45.80)**
Family history of epilepsy	Yes	8 (1.1)	53 (7.2)	Referent	Referent
	No	190 (26.0)	481 (65.7)	2.62 (1.2, 5.61)	1.48, (0.59, 3.70)
Family mental illness	Yes	13 (1.8)	63 (8.6)	Referent	Referent
	No	185 (25.3)	471 (64.3)	1.91 (1.02, 3.54)	1.05,(0.52, 2.12)
Stigma related to epilepsy	Yes	128 (17.5)	219 (29.9)	2.63 (1.88, 3.7)	1.90,(1.28, 2.80)**
	No	70 (9.6)	315 (43.0)	Referent	Referent
Age	18- < =28 years	62 (8.5)	254 (34.7)	1.54 (0.75,3.17)	2.60,(1.13, 5.94)*
	29- < =38 years	108 (14.8)	123 (16.8)	5.53 (2.71, 11.3)	5.94 (2.63, 13.42)**
	39- < =50 years	18 (2.5)	94 (12.8)	1.21 (0.52, 2.78)	1.22 (0.49, 3.03)
	> 50 years	10 (1.4)	63 (8.6)	Referent	Referent
Attitude	Unfavorable	140 (19.1)	238 (32.5)	3.02 (2.12, 4.26)	2.32,(1.56, 3.44)**
	favorable	58 (7.9)	296 (40.4)	Referent	Referent

*significant variable (*p*-value < 0.01), ** statistically significant variables (*p*-value < 0.05)

n(%) = frequency and percentage

Accordingly, those who could not read and write [AOR = 6.34, (95% CI: 2.66, 15.10)], no history of living with epileptic patients [AOR = 10.15, (95% CI: 2.25, 45.80)], age range of 18–28 [AOR = 2.60 (95% CI: 1.13, 5.94)], age groups of 29–39 [AOR = 5.94, (95% CI: 2.63, 13.42)], unfavorable attitude [AOR = 2.32, (95% CI, 1.56, 3.44)], and epilepsy related stigma [AOR = 1.90, (95% CI, 1.28, 2.80)] were variables found statistical significant with poor knowledge towards epilepsy (Table 4).

### Factors associated with attitude towards epilepsy among rural geode zone residents

In multivariate binary logistic regression analysis, stigma related to epilepsy, poor knowledge, age and perceiving that epilepsy as a God punishment for a sin were variables statistically associated with unfavorable attitude.

Participants with poor knowledge [AOR = 1.78, (95% CI: 1.18, 2.69)], perceived epilepsy stigma [AOR = 1.54, (95% CI: 1.11, 2.15)], age range of 29–39 [AOR = 2.14, (95% CI: 1.45, 3.16)], age > 50 [AOR = 2.35, (95% CI: 1.34, 4.14)] and considering epilepsy as a God punishment for a sin [AOR = 1.81, (95% CI: 1.28, 2.52)], were variables significantly associated with unfavorable attitude among residents of southern Ethiopia (Table 5).

**Table 5:** Bivariate and multivariate logistic regression analysis of associated factors with Attitude towards Epilepsy among rural residents in Gedeo zone SNNPR, Ethiopia, 2019 (*n* = 732)

Explanatory variables		Attitude		COR, (95%CI)	AOR, (95%CI)
		Unfavorable n(%)	Favorable n(%)		
Educational status	Cannot read and write	118 (16.1)	78 (10.7)	3.36, (1.80, 6.28)	1.77,(0.86, 3.64)
	Primary	152 (20.8)	124 (16.9)	2.74, (1.49, 4.99)	1.72,(0.87, 3.40)
	Secondary	67 (9.2)	84 (11.5)	1.77, (0.93, 3.37)	1.71,(0.85, 3.42)
	Preparatory	23 (3.1)	28 (3.8)	1.83, (0.83, 4.00)	1.40,(0 .60, 3.29)
	College and above	18 (2.4)	40 (5.5)	Referent	Referent
Knowledge about epilepsy	Good	238 (32.5)	296 (40.4)	Referent	Referent
	Poor	140 (19.1)	58 (7.9)	3.01 (2.12, 4.26)	1.78 (1.18, 2.69)**
living with epileptic patients	Yes	17 (2.3)	32 (4.4)	Referent	Referent
	No	361 (49.3)	322 (44.0)	2.11 (1.15, 3.87)	1.71,(0.90, 3.27)
God punishment for sin	Yes	168 (22.9)	106 (14.5)	1.87 (1.38, 2.54)	1.81,(1.28, 2.52)**
	No	210 (28.7)	248 (33.9)	Referent	Referent
Stigma related to epilepsy	Yes	199 (27.2)	148 (20.2)	1.55 (1.16, 2.07)	1.54,(1.11, 2.15)*
	No	179 (24.5)	206 (28.1)	Referent	Referent
Age	18–28	128 (17.5)	188 (25.7)	Referent	Referent
	29–39	152 (20.8)	79 (10.8)	2.83 (1.99, 4.02)	2.14,(1.45, 3.16)**
	40–50	54 (7.4)	58 (7.9)	1.37 (0.89, 2.11)	1.30 (0.80, 2.11)
	> 50	44 (6.0)	29 (4.0)	2.23 (1.33, 3.75)	2.35 (1.34, 4.14)**
Monthly Income (ETB)	<1539ETB	300 (41)	238 (32.5)	1.87 (1.34, 2.62)	1.46,(0.99, 2.16)
	≥1539ETB	78 (10.7)	116 (15.8)	Referent	Referent

* = *p*-value< 0.01, ** = *p*-value< 0.05

n(%) = frequency and percentage

## Discussion

In Ethiopia, there are different studies reporting epilepsy as a major problem within the country [8, 17]. The knowledge and attitude of people regarding epilepsy is not well addressed and even the available studies are mainly focused on college students, health professionals, teachers and medical students. Therefore, this study was intended to address this gap by assessing the knowledge and attitude of rural community residents towards epilepsy in Ethiopia.

The current study showed that about 27.0% participants had poor knowledge and 51.6% had unfavorable attitude towards epilepsy. Regarding the level of poor knowledge, the finding was lower than studies in Nigeria 43–50% [26, 27], Sudan47% [28] and north Thailand 80–99 [29]. However, our finding showed a higher magnitude of poor knowledge than the institutional study conducted in Ethiopia 14.3% [17]. The possible reason for the variation might be the difference in study design, socio cultural characteristics of the participants and study period. The other possible justifications for the difference might be the variation in study setting and populations in which the other study was conducted among students as they are more nearby for health information regarding epilepsy which attributes to decrease the magnitude of poor knowledge than community residents. Furthermore, indigenous beliefs or myths regarding epilepsy are usually common among less/none educated and community residents which can be a reason for the difference for the level of knowledge in between a group of population.

On the other hand, 51.6% of respondents reported unfavorable attitude. The finding was consistent with studies conducted in north Thailand which concludes negative attitude ranged 4–90% [29], Nigeria 55% [27], and Ethiopia in which about 51.6% belief epilepsy is a kind of insanity [17]. However, it was lower than studies conducted in Nigeria 100% respondents would not allow marriage of epileptic patients [27], Nigeria 87.2% would not employ epileptic patients, but it was higher than study done in Ethiopia 35.5% [8], china 42.1% [30], Jordan 9% [31],Vietnam (33%) [32] and Egypt (8%) [33]. The possible reason might be most of the populations in developing countries are marginalized in society as a result of negative public attitudes towards Epilepsy due to stigmas which is prevalent in cultures [32]. Another explanation might be developing countries most of the time consider the cause and treatment option for epilepsy are connected to natural methods and traditional perspectives [34]. It might be also due to difference in study area, in which most of previously done studies including our country were limited to institutions such as colleges and universities, but current study is done in previously undressed rural area on knowledge and attitude towards this commonly occurring neurological problem in our country.

The second objective of our study was to identify factors associated with poor knowledge and unfavorable attitudes. Participants who could not read and write were more likely to have poor knowledge as compared with college and above graduates and this was supported by study conducted in Jordan [31], systematic review study done in different countries [35–39] and other study done in Nigeria [40], Sudan [28] and Ethiopia [8]. The possible explanation might be due to the difference in the educational level which affects their ability to understand and perceive epilepsy.

Other factors which were associated with poor knowledge were absence of history of living with epileptic patient, poor attitude, stigma related to epilepsy and age range of 18–28 and 29–36. This finding was also supported by study in Ethiopia [8] and other systematic study conducted from Asian countries in which individual who have no experience of contact with epilepsy have poor knowledge [13].

Poor knowledge, stigma related to epilepsy, considering the cause of epilepsy as God punishment for sin and being elder were associated with unfavorable attitude towards epilepsy. It was in agreement with studies conducted in Ethiopia [8], Ismailia [41], Malaysia, UK and Thailand [13, 35–37]. The possible reason to this might be people with epilepsy are marginalized in society as a result of negative public attitudes towards Epilepsy. Epilepsy stigma, which is prevalent in cultures and backward laws in many places throughout the world has also negative consequences in developing countries including Ethiopia [32]. In Ethiopia, elderly population living in rural communities have still limited access to education which might lead to negative belief regarding epilepsy and most community residents believe cause of epilepsy related to punishment from the God for wrong doing.

### Limitations

The first limitation of this study might be due to the cross-sectional nature of study design which cannot establish the temporal relationship between the dependent variable and its associated factors. Secondly, since the data was collected by face to face interview method, it might prone to social desirability bias especially in case of epilepsy stigma. Thirdly, the definition of epilepsy within a community might not allow the possibility of outgrowing epilepsy, since epilepsy was not defined in clinical terms as a result what participants think and perceive to be epilepsy based on their experience might not be potentially epilepsy.

## Conclusion

There is a gap regarding the knowledge and attitude towards epilepsy among southern Ethiopia community residents. This demonstrates a need for community educational program regarding epilepsy which can increase community awareness particularly in rural areas to decrease stigma and negative beliefs towards epilepsy. Home visit and education focusing on epilepsy should be applied by health extension workers.

## Data Availability

All raw data included in the manuscript can be accessed from the corresponding author through the email address of “alexmolla09@gmail.com” with rational request.

## Abbreviations

AOR: Adjusted Odds Ratio
CI: Confidence Interval
COR: Crude Odds Ratio
PLWE: People Living With Epilepsy
SNNRE: Southern Nation Nationalities and Regions of Ethiopia
WHO: World Health Organization
